# Features of alpha-synuclein that could explain the progression and irreversibility of Parkinson's disease

**DOI:** 10.3389/fnins.2015.00059

**Published:** 2015-03-09

**Authors:** Scarlet Gallegos, Carla Pacheco, Christian Peters, Carlos M. Opazo, Luis G. Aguayo

**Affiliations:** ^1^Laboratory of Neurophysiology, Department of Physiology, University of ConcepcionConcepcio, Chile; ^2^Oxidation Biology Laboratory, The Florey Institute of Neuroscience and Mental Health, University of MelbourneMelbourne, VIC, Australia

**Keywords:** Parkinson's disease, alpha-synuclein, transmission, toxicity, neurodegeneration

## Abstract

Alpha-synuclein is a presynaptic protein expressed throughout the central nervous system, and it is the main component of Lewy bodies, one of the histopathological features of Parkinson's disease (PD) which is a progressive and irreversible neurodegenerative disorder. The conformational flexibility of α-synuclein allows it to adopt different conformations, i.e., bound to membranes or form aggregates, the oligomers are believed to be the more toxic species. In this review, we will focus on two major features of α-synuclein, transmission and toxicity, that could help to understand the pathological characteristics of PD. One important feature of α-synuclein is its ability to be transmitted from neuron to neuron using mechanisms such as endocytosis, plasma membrane penetration or through exosomes, thus propagating the Lewy body pathology to different brain regions thereby contributing to the progressiveness of PD. The second feature of α-synuclein is that it confers cytotoxicity to recipient cells, principally when it is in an oligomeric state. This form causes mitochondrial dysfunction, endoplasmic reticulum stress, oxidative stress, proteasome impairment, disruption of plasma membrane and pore formation that lead to apoptosis pathway activation and consequent cell death. The complexity of α-synuclein oligomerization and formation of toxic species could be a major factor for the irreversibility of PD and could also explain the lack of successful therapies to halt the disease.

## General overview

Misfolded proteins are rich in β-sheet structure with a high tendency to form long fibrillar aggregates known as amyloid deposits (Soto, 2012). Amyloid is a generic term referring to organized protein aggregates with specific staining properties, higher resistance to proteolytic degradation and a fibrillar appearance when observed with electronic microscopy (Soto, 2003). β-structures are harder to degrade than α-helixes (main conformation of native proteins), which explains why amyloid deposits cannot be removed by the proteasome system (Lee and Yu, 2005). The aggregation of misfolded proteins is believed to occur when hydrophobic residues exposed at the surface of proteins interact with other misfolded proteins (Doyle et al., 2011).

There is a group of neurodegenerative diseases characterized by the misfolding of proteins into β-sheet aggregated structures that accumulate as amyloid deposits in affected tissues (Moreno-Gonzalez and Soto, 2011). These conformational disorders or protein misfolding diseases include Alzheimer's disease (AD) with extracellular plaques of amyloid-β protein (Aβ) and tangles of hyperphosphorylated tau protein in the cytoplasm of neurons; Parkinson's disease (PD) where the cytoplasm of neurons in the substantia nigra contains aggregates called Lewy bodies composed principally of the protein α-synuclein (α-syn); Huntington's disease; Amyotrophic Lateral Sclerosis; and transmissible spongiform encephalopathy characterized by an accumulation of aggregates of the prion protein (PrP) in the brain (Soto, 2003).

Prion proteins propagate by auto-catalytic conversion of native, nonpathogenic forms of the protein (PrP^C^) expressed in several types of human cells (Frost and Diamond, 2010), into misfolded pathological conformation (PrP^Sc^) (Moreno-Gonzalez and Soto, 2011; Costanzo and Zurzolo, 2013). In prion disease, the pathological conformation is the primary infectious agent (Soto and Satani, 2011; Munch and Bertolotti, 2012) that propagates using a mechanism called “seeding/nucleation” where PrP^Sc^ acts like a seed that recruits and converts soluble protein into aggregates of PrP^C^ which form polymers (Moreno-Gonzalez and Soto, 2011; Munch and Bertolotti, 2012). As prions grow and spread, they interfere with the function of the nervous system resulting in progression of the disease in affected patients (Jucker and Walker, 2013).

Accumulating experimental data indicate that the seeding principle, similar to the conversion of PrP^C^ to PrP^Sc^ in prion diseases, also applies to other proteins associated to neurodegenerative diseases such as AD and PD (Soto, 2012; Costanzo and Zurzolo, 2013; Jucker and Walker, 2013). In this review, we will address features of the α-syn protein that propagates and causes toxicity in recipient neurons, which could explain the progressive and irreversible pathology of PD.

## Parkinson's disease

Parkinson's disease (PD) is the second most common neurodegenerative disease after AD (Feng et al., 2010). The two pathological hallmarks of PD are the presence of cytoplasmic inclusions termed Lewy bodies and Lewy neurites, and the selective degeneration and loss of dopaminergic neurons in the substantia nigra pars compacta (SNpc) (Smith et al., 2005; Luk et al., 2012a) leading to the main motor symptoms of PD, i.e., resting tremor, muscle rigidity, bradykinesia, and postural instability (Jankovic, 2008; Feng et al., 2010; Luk et al., 2012b). For this reason, PD is generally defined as a movement disorder associated with degeneration of neurons in the nigrostriatal system (Desplats et al., 2009). The diagnosis of PD relies mainly on the clinical detection of these motor symptoms, but there is no definitive diagnostic test. The symptoms are often accompanied by the appearance of autonomic, cognitive and psychiatric problems (Mercuri and Bernardi, 2005; Jankovic, 2008).

During the early stages of the disease, called the presymptomatic phase, PD patients develop non-motor deficits including olfaction impairment, vagal dysfunction and sleep disorders (Angot and Brundin, 2009). The typical motor symptoms appear when there is 50–60% of dopaminergic neuron loss and 70–80% of dopamine depletion (Schapira, 2009; Sato et al., 2013). The cognitive functions decline at more advanced stages (Angot and Brundin, 2009). As a result of these clinical symptoms, PD is now recognized as a complex clinicopathological entity. The presence of cognitive impairment or dementia in patients with PD is associated with loss of independence, a lower quality of life and a reduction in survival time (Irwin et al., 2013). The majority of PD cases are sporadic (Feng et al., 2010) and the etiology and pathogenesis remain enigmatic (Brundin et al., 2008). The origin and development of the disease seems to involve both genetic susceptibility and environmental factors such as oxidative stress, proteasome inhibition, and aging (Ross and Poirier, 2004; Smith et al., 2005).

### Pathological findings

The pathological hallmark of PD is the presence of intraneuronal proteinaceous inclusions called Lewy bodies (LBs) or Lewy neurites (LNs) depending if they are localized in the cell body or the processes, respectively (Angot and Brundin, 2009). LBs are spherical eosinophilic cytoplasmic protein aggregates that contain ubiquitin and fibrils of α-syn, located in the substantia nigra and in several central nervous system structures (Bisaglia et al., 2009; Soto, 2012). The main protein component of LBs is α-syn, a synaptic protein with the propensity to misfold and aggregate (Angot et al., 2012). Analysis of these inclusions with immunohistochemistry techniques revealed that they have affinity to specific dyes that identify misfolded α-syn, such as Thioflavin S that recognizes β-sheet conformations, and for α-syn phosphorylated on serine 129 (Ser129), which is a post-translational modification that only occurs in LBs (Angot and Brundin, 2009). In PD, the accumulation of Lewy bodies undergo an ascending pattern of progression, spreading from the lower brainstem and olfactory bulb into the limbic system and, eventually, to the neocortex, suggesting a propagation mechanism similar to prion diseases (Desplats et al., 2009).

### PD is progressive and irreversible

There are two major features that identify PD: (1) progressive damage of dopaminergic neurons in the SNpc that leads to the depletion of dopamine (DA) release necessary to maintain essential functions (Mercuri and Bernardi, 2005), and (2) the appearance of non-motor symptoms associated to the degeneration of non-dopaminergic systems (Obeso et al., 2010) together with propagation and accumulation of Lewy bodies in different brain regions (Ross and Poirier, 2004; Desplats et al., 2009; Obeso et al., 2010) making PD an irreversible, and at the present, an incurable disorder (Singh et al., 2007).

Current pharmacological treatment for PD involves the use of the dopamine precursor L-3,4-dihydroxyphenylalanine (L-dopa) which alleviates bradykinesia, the increase in muscle tone and tremor, but does not reduce non-motor symptoms (Mercuri and Bernardi, 2005). DA agonists, COMT, and MAO-B inhibitors are also used as adjunctive treatment to L-dopa (Schapira, 2009; Tarazi et al., 2014). In addition, the efficiency of L-dopa decreases over time and many patients develop motor fluctuations (wearing-off and on–off phenomena), dyskinesias and behavioral abnormalities (Mercuri and Bernardi, 2005; Singh et al., 2007; Obeso et al., 2010). Newer non-pharmacological therapies include the use of viral vector genes to silence defective genes associated with PD. Surgical interventions such as deep brain stimulation, pallidotomy, and thalamotomy, or non-invasive procedures such as gamma knife radiation are also employed (Tarazi et al., 2014). The available pharmacological and non-pharmacological treatments focus primarily on motor symptoms, but do not modify the progression of DA neuronal degeneration in the SNpc or the other affected areas (Mercuri and Bernardi, 2005; Singh et al., 2007; Tarazi et al., 2014).

## Alpha-synuclein

### Structure

Alpha-synuclein (α-syn) is a small, acidic protein of 14.5 kDa and 140 amino acids (Bisaglia et al., 2009) that is highly conserved in vertebrates and is expressed in presynaptic nerve terminals in several regions of the brain (Jain et al., 2013). The protein belongs to the synuclein family, α-, β- and γ-synuclein, from three highly expressed human genes (SNCA, SNCB, and SNCG) (Lavedan, 1998). The β-isoform also exhibits a presynaptic location and co-localizes with α-syn at many, but not all presynaptic terminals, whereas γ-synuclein is expressed by glia cells and specific neuronal populations, mainly dopamine neurons (Bendor et al., 2013). α-syn lacks a defined secondary structure and therefore belongs to the intrinsically unstructured protein family (Bisaglia et al., 2009; Deleersnijder et al., 2013). However, the remarkable conformational plasticity of α-syn allows it to adopt a wide range of dynamic structures depending on the environment and binding partners (Jain et al., 2013).

**Figure 1:**
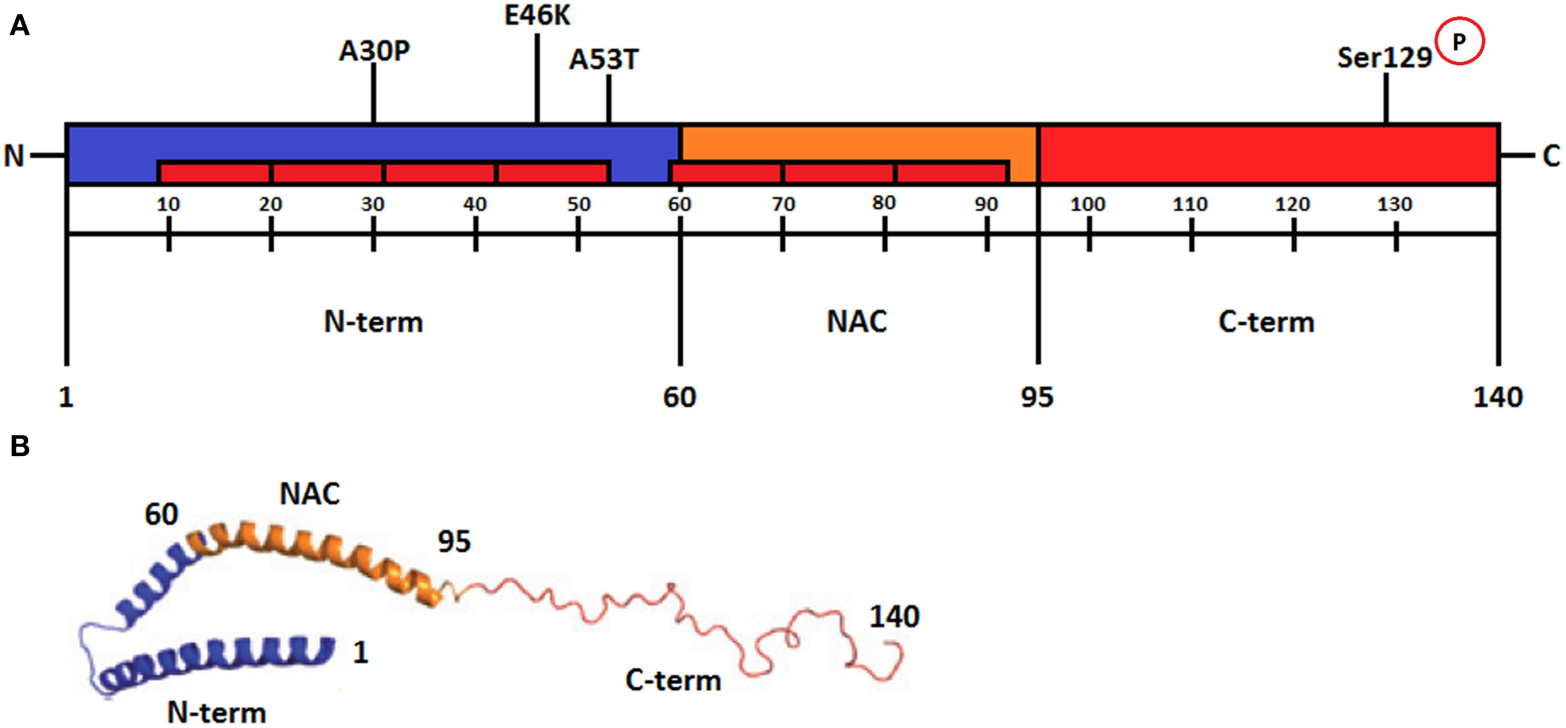
**(A)** Schematic representation of α-synuclein regions. The amino-terminal from amino acids 1–60 is an amphipathic region responsible for α-syn-membrane interactions. It contains repeats of hexamer motifs similar to apolipoproteins (represented in the figure as red rectangles). The point mutations of α-syn are located in this region (A30P, E46K, and A53T). The central region from amino acids 61-95, termed NAC (non-β amyloid component), is the most hydrophobic portion of the protein and is required for the aggregation process. This region folds into a β-sheet secondary structure and forms amyloid fibrils. The C-terminal from amino acids 96–140 is characterized by the presence of acidic residues and several negative charges. The residue serine 129 in this region is phosphorylated in Lewy bodies (Modified from Plotegher et al., 2014). **(B)** Schematic representation of micelle-bound α-synuclein. The N-terminal region with antiparallel α-helices is shown in blue, the NAC region is also an α-helix and is shown in orange, and the unstructured C-terminal part is shown in red. Numbers refer to amino acid residues. (Modified from Lashuel et al., 2013). Reprinted by permission from MacMillan Publishers Ltd (Nature Reviews Neuroscience).

Figure 1A illustrates the three distinct regions of α-syn. (1) The amino-terminal sequence of α-syn (residues 1–60) contains variants of an imperfect 11 amino acid repeat with a highly conserved hexamer motif similar to that found in the amphipathic helices of apolipoproteins (Bellucci et al., 2012; Deleersnijder et al., 2013). This portion of the protein includes the A30P, A53T, and E46K mutation sites found in familial PD cases (Bisaglia et al., 2009). (2) The central region (residues 61–95) termed NAC (non-amyloid-β component) contains two additional motifs and is the most hydrophobic portion of the protein. This region can undergo a conformational change from a random coil to β-sheet structure and is able to form cylindrical β-sheets and amyloid-β-like fibrils (Bellucci et al., 2012; Deleersnijder et al., 2013). (3) The carboxy-terminal of α-syn (residue 96–140) is rich in acidic residues (Bisaglia et al., 2009) and is responsible for the intrinsically disordered nature of α-syn. This region also plays a regulatory role in the aggregation and fibril formation of the protein (Deleersnijder et al., 2013). Dopamine can interact nonspecifically with the C-terminal residues of α-syn, suggesting that inappropriate C-terminal cleavage of this protein, which is known to occur in PD brains, might affect DA homeostasis (Bisaglia et al., 2009; Bellucci et al., 2012).

Phosphorylation is one of the most common posttranslational modifications and has a role on the function of numerous target proteins (Sato et al., 2013). Thus, α-syn phosphorylated in Ser129 (pS129-α-syn) may have some functions in the normal brain. Residue Ser129 in the C-terminal region of α-syn is approximately 90% phosphorylated in Lewy Bodies (Figure 1A). This form is biochemically detectable in cerebral cortex, substantia nigra and nucleus basalis of Meynert in normal human subjects (Walker et al., 2013). Interestingly, no more than 4% of α-syn is phosphorylated at this residue in normal brain suggesting a strict control. The phosphorylation of Ser129 is controlled by kinases, phosphatases, and degradation pathways (Hara et al., 2013; Sato et al., 2013). Several studies identified the kinases responsible for the phosphorylation of α-syn *in vitro* and *in vivo*, such as Polo-like kinase PLK2 and PLK3, casein kinase CK1 and CK2 and members of the G protein-coupled receptor kinase family GRK2, GRK3, GRK5, and GRK6 (Hara et al., 2013; Sato et al., 2013; Kosten et al., 2014). Phosphoprotein phosphatase 2A (PPA2) might be responsible for dephosphorylation of α-syn, and stimulating the activity of PP2A resulted in reduced levels of pS129-α-syn and aggregated α-syn (Sato et al., 2013; Walker et al., 2013).

*In vitro* studies suggest that α-syn interacts with lipid membranes through its repeat motifs and with small unilamellar vesicles and micelles preferentially containing negatively charged head groups (Ulmer et al., 2005). When α-syn interacts with vesicles or membranes, the N-terminal of the protein acquires an α-helical conformation that can be an extended helix or antiparallel helices connected by a short linker, depending on the membrane properties. The C-terminal of the protein remains unstructured, as observed in Figure 1B (Ulmer et al., 2005; Plotegher et al., 2014). The binding of α-syn to phospholipid micelles produces a flattening in their surface curvature. In addition, the α-syn mutants A53T and E46K exhibit increased membrane-binding affinity and also flatten micelle surface curvature, whereas the A30P α-syn mutant has a decreased membrane affinity and does not alter the surface curvature of the micelles (Auluck et al., 2010).

### Physiological roles

Studies suggest that α-synuclein is involved in the control of synaptic membrane processes (Bellucci et al., 2012) and participates in the control of neurotransmitter release via interactions with members of the SNARE family (Tsigelny et al., 2012). More specifically, α-syn promotes SNARE-complex assembly through a non-enzymatic mechanism, binding to phospholipids via its N-terminal and to synaptobrevin-2 via its C-terminal (Burre et al., 2010). However, the precise physiological functions of α-syn remain uncertain and studies are often conflicting (Luk et al., 2009). Individual synuclein knockout animals (α-, β- or γ-syn) are viable (Lashuel et al., 2013) and results obtained in α-syn knockout mice suggest that α-syn is not essential for synapse formation or cell survival (Bisaglia et al., 2009) as supported by the relatively late translocation of α-syn into presynaptic terminals during synaptogenesis (Chandra et al., 2004).

The existence of three synuclein isoforms and the high degree of co-expression of α- and β-syn raises the possibility of redundant synaptic functions (Chandra et al., 2004; Greten-Harrison et al., 2010). Analysis of α-/β-synuclein double knockout mice did not show major changes in synaptic functions such as neurotransmitter release, synaptic vesicle numbers, or synaptic plasticity, and caused no structural abnormalities in the overall brain morphology. However, the α-/β- double knockout did show a modest reduction in striatal DA levels (Chandra et al., 2004; Bendor et al., 2013), and it has been reported that α-syn binds to C-terminal of the DA transporter increasing the DA uptake (Hara et al., 2013). The triple synuclein knockout mice (TKO) were viable and fertile, but developed severe neurological impairments resulting in a striking age-dependent survival deficit ending in premature death (Burre et al., 2010; Greten-Harrison et al., 2010). Furthermore, the TKO mice exhibited an age-dependent decrease in SNARE-complex assembly. Thus, synucleins are required for maintaining normal SNARE-complex assembly during aging in mice (Burre et al., 2010), and they are also relevant for long-term survival (Greten-Harrison et al., 2010).

### Pathological roles

The pathological roles of α-syn arise from the intracellular accumulation of α-syn amyloid fibrils that define a family of neurological disorders termed α-synucleinopathies in which PD is included (Luk et al., 2009; Danzer et al., 2012). α-syn is able to form oligomers, fibrils and large aggregates following overexpression, exposure to changes in pH, oxidative stress, or by interaction with dopamine (Feng et al., 2010). Unlike α-syn, β-syn does not fibrillize and both β- and γ-syn can inhibit the aggregation of α-syn *in vitro* and *in vivo* (Bendor et al., 2013). Several post-translational covalent modifications of α-syn have been described to promote the pathological changes in the protein, including serine and tyrosine phosphorylation, ubiquitination, nitration and C-terminal truncation (Ross and Poirier, 2004; Deleersnijder et al., 2013). The predominant modification of α-syn in Lewy bodies is single phosphorylation at Ser129. However, the relationship between Ser129 phosphorylation and α-syn aggregation remains unclear (Sato et al., 2013). Histopathological studies of human brain show that increased levels of pS129-α-syn are present in soluble and detergent insoluble brain fractions of Lewy Body pathology. Thus, phosphorylation and ubiquitination presumably increase aggregation and formation of an insoluble fraction. As the α-syn solubility decreases, phosphorylation seems to increase even more (Walker et al., 2013).

Mutations in the gene encoding α-syn (SNCA) directly link α-syn with the onset of PD (Plotegher et al., 2014). The overexpression of α-syn due to duplication or triplication of the SNCA gene causes rare familial forms of parkinsonism, and single nucleotide polymorphisms in the SNCA gene (Ala53Thr, Ala30Pro, and Glu46Lys) are linked to sporadic PD (Angot et al., 2012). Recently, new mutations have also been found in the N-terminal part of the protein (Deleersnijder et al., 2013). The three most common PD-related point mutations have shown to accelerate α-syn aggregation (but not necessarily fibril formation) *in vitro*. These effects can be explained by changes in net charge, hydrophobicity, and secondary structure propensity (Deleersnijder et al., 2013). This suggests that α-syn is heavily implicated in the pathogenesis of PD, both familial and sporadic cases (Smith et al., 2005; Angot et al., 2012).

### Critical features of α-syn favoring its transmission and neurotoxicity

#### Cellular transmission

Autopsy reports of PD patients who received bilateral intrastriatal grafts from healthy embryonic mesencephalic dopaminergic neurons, 10–22 years before the postmortem analysis (Li et al., 2008), showed that some of the grafted neurons contained pathological α-syn or Lewy bodies similar to those observed in the host brain (Li et al., 2008; Angot et al., 2012). These studies reported that the LB and LN developed in the grafted neurons were ubiquitinated, positive for Thioflavin S staining, and that α-syn was phosphorylated on Ser129 indicating that it was aggregated, post-translationally modified and disease-related (Brundin et al., 2008; Li et al., 2008; Angot and Brundin, 2009). It was also found that the α-syn accumulation in grafted neurons was time-dependent (Brundin et al., 2008). The presence of pathology in the grafted neurons could have been triggered by misfolded α-syn in the host brain, which was transmitted into grafted cells with subsequent seeding of aggregates in the recipient cells (Li et al., 2008). These findings suggest that α-syn pathology propagates by a mechanism similar to prion diseases (Hansen et al., 2011). For this reason, numerous studies have attempted to replicate and explain these findings in cell and animal models (Angot et al., 2012).

The introduction of exogenously assembled α-syn fibrils to various cells overexpressing α-syn catalyzed intracellular α-syn aggregation. The α-syn fibrils recruited endogenous soluble α-syn, converting them into misfolded detergent-insoluble inclusions which were hyperphosphorylated and ubiquitinated, resembling Lewy bodies (Luk et al., 2009). In another study, a specific type of α-syn oligomer was capable of inducing α-syn seeding and aggregation after exogenous application to a neuroblastoma cell line and primary cortical neurons. This aggregation process was dose- and time-dependent (Danzer et al., 2009). An *in vivo* study showed that human α-syn expressed in mice was transmitted from cells in the brain to dopaminergic neurons grafted into the striatum, in analogy to the mechanism suggested to take place in the grafted PD cases previously mentioned (Angot et al., 2012). The presence of Lewy bodies in grafted neurons was a time-dependent process, with a higher percentage of neurons displaying Lewy bodies in older grafts than in younger transplants (Angot et al., 2012).

Studies with injected homogenates prepared from brainstem and spinal cord of aged symptomatic animals that contained abundant LB-like pathology, or with preformed fibrils (PFF) assembled from human α-syn, into the neocortex and striatum (Luk et al., 2012a) showed that despite the fact that inoculations were unilateral, intraneuronal α-syn deposits were widely distributed bilaterally and present throughout the CNS. This indicated that α-syn pathology expanded through the CNS in a time-dependent manner and accelerated the disease *in vivo* (Luk et al., 2012b). Another study with non-transgenic mice (WT) that were injected unilaterally with PFFs of α-syn in the striatum showed that deposits of hyperphosphorylated α-syn were visible at the injection site and several areas directly interconnected to the striatum (Luk et al., 2012b). The contralateral neocortex also developed LBs, revealing once again a time-dependent dissemination of these inclusions. The striatal inoculation led to abundant α-syn pathology in neurons located in the SNpc. This was accompanied by the gradual loss of tyrosine hydroxylase immunoreactivity, suggesting that intraneuronal α-syn inclusions lead to neurodegeneration and DA neuronal loss. In summary, this study demonstrated that a single intrastriatal injection of synthetic misfolded α-syn into WT mice induced a neurodegenerative process, with accumulation of intracellular LB/LN pathology, selective loss of SNpc neurons, and impaired motor coordination (Luk et al., 2012a).

#### Cellular transmission mechanisms

Lewy Body propagation within the CNS could be associated with cell-to-cell transmission that involves the release of misfolded α-syn from donor cells into the extracellular space. Recipient cells could then take up the misfolded α-syn which recruits native unfolded α-syn from the cytosol of the recipient cell and acts as a template for the development of aggregates that eventually lead to the formation of Lewy Bodies (Angot and Brundin, 2009; Steiner et al., 2011). Studies have shown that cells can release α-syn into their surroundings. For example, nanomolar concentrations of extracellular monomeric and oligomeric α-syn have been detected in cerebrospinal fluid and plasma samples from both PD patients and controls (El-Agnaf et al., 2006; Bellucci et al., 2012; Costanzo and Zurzolo, 2013), with a significantly higher concentration in PD patients (El-Agnaf et al., 2006) supporting the occurrence of a secretory process for α-syn (Bellucci et al., 2012).

**Figure 2:**
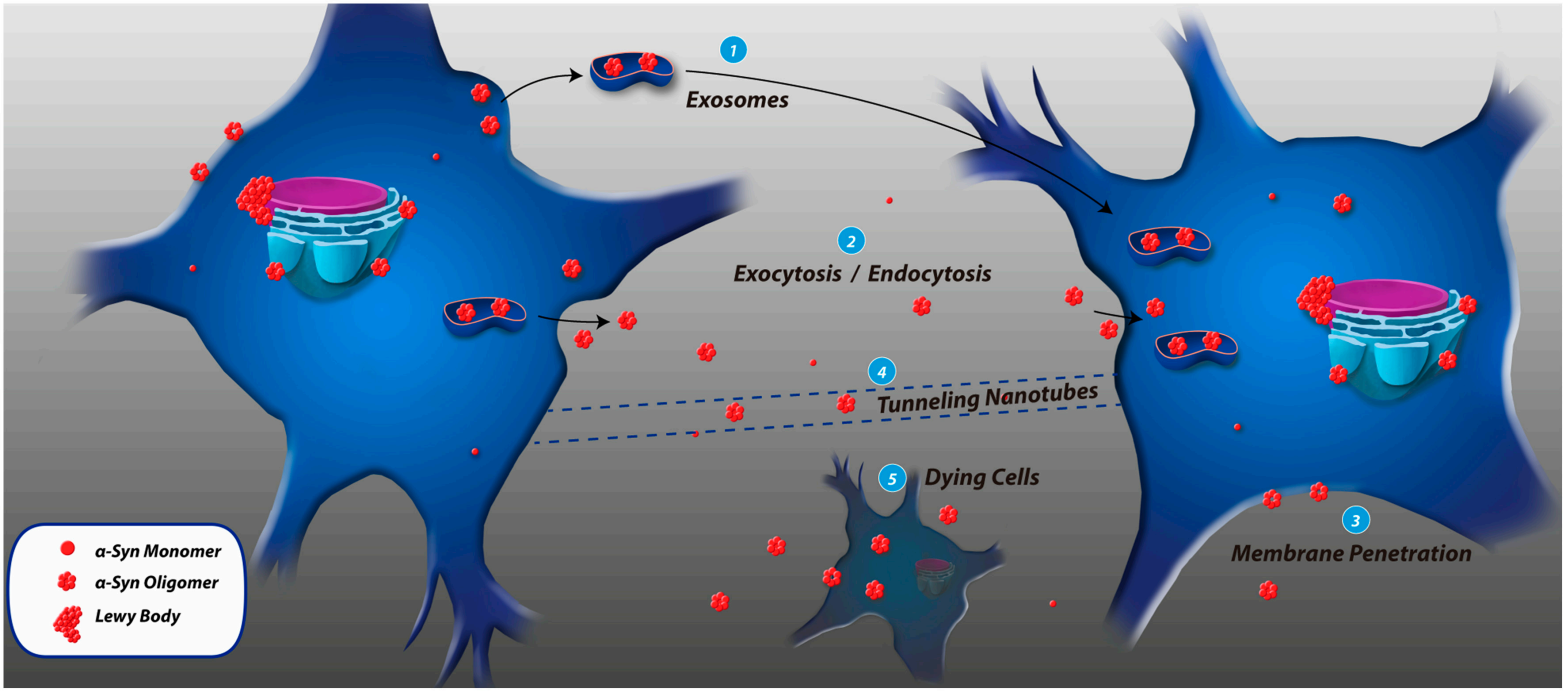
**Cell-to-cell transmission mechanisms of α-synuclein**. **(1)** Exosomes are small membrane vesicles derived from the endocytic pathway, and the presence of α-syn inside exosomes and its transmission to recipient cells has been reported. **(2)** α-syn could be released through an exocytosis process and accumulate in the extracellular space and be taken up by other cells through endocytosis. **(3)** α-syn associates to biological membranes by its N-terminal, penetrates the membrane and gains access to the cytosol. **(4)** Tunneling nanotubes are membrane bridges of actin between cells, but this mechanism has not been identified to participate in α-syn transmission. **(5)** Dying cells could be an important reservoir of pathological α-syn when they eventually expel their contents after being lysed.

Several transmission mechanisms have been reported for α-syn (see Figure 2). For example, SH-SY5Y cells release α-syn and its mutant variants A53T and A30P into the extracellular space. The secretion is considerably reduced at 18°C, suggesting that α-syn is released from these cells via exocytosis (Lee et al., 2005). However, Brefeldin A, a classical inhibitor of ER/Golgi-dependent secretion, does not block α-syn release indicating that α-syn exocytosis relies on an unconventional secretory pathway (Lee et al., 2005; Angot and Brundin, 2009; Costanzo and Zurzolo, 2013). Neuronal cells subjected to various stress conditions including oxidative stress and proteolytic impairment increase the translocation of α-syn into vesicles and the release of monomeric and aggregated α-syn (Jang et al., 2010). Failure of intracellular clearance pathways, such as autophagy, could also contribute to the pathological release of α-syn increasing its transfer to other cells (Lee et al., 2013). The release of pathogenic α-syn from dying cells [see Figure 2(5)] could also contribute to the extracellular pool of α-syn in the brain (Angot and Brundin, 2009). It has been reported that α-syn moves antero- and retrogradely along axons (Steiner et al., 2011). For example, α-syn fibrils could be internalized, anterogradely transported within axons, released, and subsequently taken up by additional neurons (Freundt et al., 2012). Recently, tunneling nanotubes (TNTs) have been proposed to be involved in intercellular propagation of PrP^Sc^. As observed in [Figure 2(4)], TNTs are actin-containing membrane bridges between cells (Angot and Brundin, 2009) acting as conduits for the exchange of cytosolic and membrane-bound molecules and organelles, as well as for the spreading of pathogens (Costanzo and Zurzolo, 2013). TNTs have not yet been implicated in other neurodegenerative diseases, or the spreading of other misfolded proteins such as α-syn (Angot and Brundin, 2009).

***Membrane penetration***. Several studies have shown that the 11-amino acid imperfect repeats of α-syn play a critical role in the cell membrane translocation of α-syn. This uptake process is not inhibited when Chinese hamster ovary cells were incubated at a low temperature (4°C), suggesting that receptor-mediated endocytosis is not involved. In addition, the uptake of α-syn is insensitive to treatment with the general exo/endocytosis inhibitors Brefeldin A and Cytochalasin D (Ahn et al., 2006). A study using computer modeling and membrane simulations investigated the course of penetration of WT and A53T mutant α-syn in the membranes and pore formation activity. Their data showed that the N-terminal initially makes contact with the surface of the membrane, undergoing changes in secondary structure, and that the penetration of the A53T mutant α-syn across the membrane was 20% faster than WT α-syn (Tsigelny et al., 2012).

***Exosomes***. Exosomes have been proposed to participate in the spreading of disease-related proteins within the brain, such as PrP^Sc^ in prion diseases and β-amyloid peptide (Aβ) in AD. Exosomes are small membrane vesicles derived from the endocytic pathway and are released from cells into the surroundings. A wide range of cells secrete exosomes *in vitro*, including neurons and astrocytes (Angot and Brundin, 2009). For this reason, Danzer et al. (2012) investigated whether oligomeric species of α-syn are present in exosomes. The authors provided evidence that α-syn oligomers are indeed present in the exosomal fractions from both neuronal and non-neuronal cells. According to their results, exosome-associated α-syn oligomers are more prone to being taken up by cells than exosome-free α-syn oligomers and confer more cytotoxicity when compared to the increase in Caspase 3/7 activation (Danzer et al., 2012).

In another study, α-syn was also detected in exosomes of cells overexpressing α-syn and transmitted to normal SH-SY5Y cells. It has been reported that lysosomal function is decreased in PD patients, and α-syn requires the lysosome for its degradation. Thus, the investigators inhibited lysosomal function in the exosome donor cells and the exosomal α-syn level increased significantly, as well as the release of α-syn which led to greater transmission to recipient cells and a greater number of cells containing α-syn inclusions. The hypothesis of this group is that lysosomal dysfunction can accelerate exosomal α-syn release and propagation to neighboring cells with associated increase in α-syn inclusion formation (Alvarez-Erviti et al., 2011).

***Endocytosis***. Another transmission mechanism of α-syn was described in a study that used human dopaminergic neuronal cells treated with α-syn. The cells take up α-syn and approximately half of the inclusions formed in these cells displayed ubiquitin immunoreactivity and were stained with thioflavin S, resembling Lewy Bodies. Dynamin-1 K44A, a dominant negative mutant that blocks endocytic vesicle formation, reduced significantly the transmission of α-syn, as compared to cells not expressing this inhibitor. In addition, 90% of the transmitted α-syn was colocalized with endosomal GTPases rab5a and rab7 to further support endocytosis as a possible transmission mechanism (Desplats et al., 2009).

A similar study reported the entry of α-syn from the conditioned medium into SH-SY5Y cells. The α-syn uptake was observed in cells incubated at 37°C, but not in cells incubated at 4°C suggesting that α-syn could be taken up by endocytosis. To validate this observation, cells were co-cultured with the endocytosis inhibitors monodansylcadaverine or dynasore. These inhibitors decreased the number of cells with α-syn inclusions, indicating that endocytosis plays an important role in the α-syn intercellular transfer. To determine whether endocytic mechanisms were also involved in the uptake of α-syn *in vivo*, dynasore was coinjected with α-syn monomers in the right neocortex of rats. The data revealed that the α-syn signal was reduced by 40% in the right cortex where the inhibitor was coinjected with α-syn, suggesting that endocytosis contributes significantly to neuronal uptake of α-syn both *in vitro* and *in vivo* (Hansen et al., 2011).

PFFs of α-syn also enter inside neurons and seed recruitment of endogenous α-syn to initiate accumulation of pathologic hyperphosphorylated α-syn. Treatment of neurons with α-syn PFFs in the presence of wheat germ agglutinin (WGA) induced adsorptive mediated endocytosis, which increased the extent of α-syn pathology in a dose-dependent manner. The addition of a competitive inhibitor of WGA reduced its effects on α-syn-induced aggregate formation. Taken together, these findings indicate that α-syn PFFs could gain access to the neuronal cytoplasm by adsorptive endocytosis (Volpicelli-Daley et al., 2011).

#### Mechanisms of neurotoxicity of α-synuclein

Under pathological conditions, progressive accumulation of α-syn and the formation of oligomers have been proposed to play a critical role in the pathogenesis of PD and other α-synucleinopathies (Tsigelny et al., 2012). However, there are still unresolved issues according to the precise mechanisms through which α-syn aggregation contributes to neurodegeneration, the nature of the toxic forms of α-syn and the cellular pathways that are affected by α-syn (Tsigelny et al., 2012; Lashuel et al., 2013).

The multifunctional properties of α-syn may lie in its conformational flexibility, which allows the protein to change its conformation upon interaction with biological membranes of different compositions, other proteins or protein complexes (Lashuel et al., 2013; Plotegher et al., 2014). Several factors including oxidative stress, long incubations at 37°C, post-translational modifications, the concentrations of fatty acids, phospholipids, and metal ions (Fe^2+^, Cu^2+^, and Zn^2+^), or application of various ligands such as dopamine have shown to induce and/or modulate α-syn structure and oligomerization *in vitro* (Lashuel et al., 2013). Numerous studies show that the soluble oligomers have a higher cytotoxicity compared to the fibrillar form of the protein (Stockl et al., 2013).

**Figure 3:**
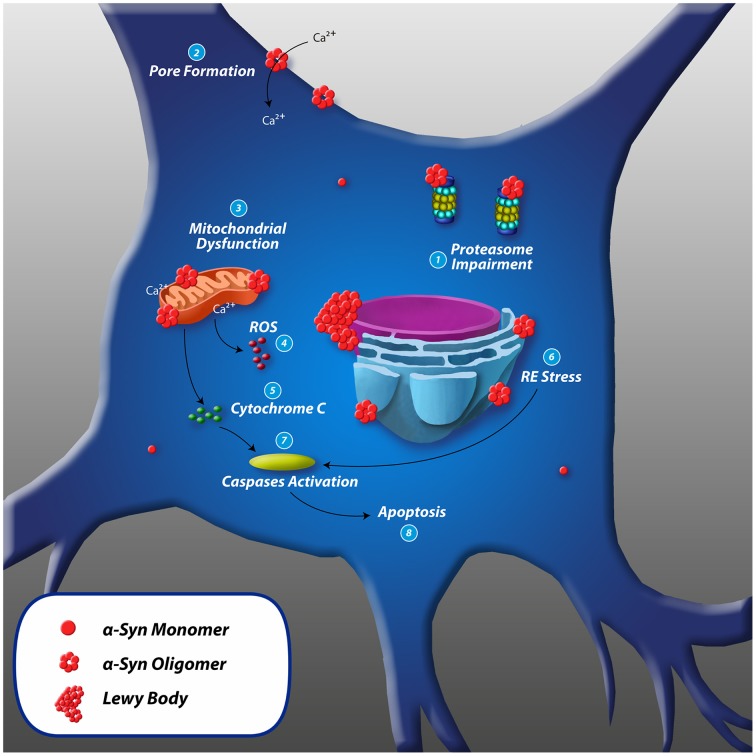
**Toxic mechanisms of α-synuclein and cell death. (1)** Proteasome impairment: inhibition of chymotryptic, tryptic and post-acidic proteasome activity leading to further intracellular accumulation of misfolded proteins such as α-syn. **(2)** Pore formation: α-syn oligomers penetrate cellular membranes increasing the conductance by forming pore-like structures that could act as non-selective channels, resulting in abnormal calcium influx. **(3)** Mitochondrial dysfunction: α-syn associates to the mitochondrial inner and outer membrane and **(4)** Increases mitochondrial and intracellular ROS levels. **(5)** Release of cytochrome C: accumulation of intramitochondrial ROS and Ca^2+^ leads to reduction in mitochondrial membrane potential and opening of permeability transition pores that could cause the release of cytochrome C to the cytosol. **(6)** RE stress: cellular accumulation of misfolded proteins can lead to chronic endoplasmic reticulum stress. α-syn associates to ER membrane and causes morphologic dysfunction such as dilated cisternae and increases the level of ER chaperones. **(7)** Cytochrome C leads to activation of caspase-3 and -9, and ER stress leads to activation of caspase-12. **(8)** Caspases initiate apoptosis leading to cell death.

Several different toxic mechanisms have been associated to the α-syn aggregation process and to its oligomeric and fibrillar products. Due to the heterogeneity of the aggregation products, it is reasonable to find a large variety of noxious effects attributed to α-syn (Plotegher et al., 2014). Similar to transmission, α-syn cytotoxicity may occur through different mechanisms. See Figure 3.

***Proteasome impairment and oxidative stress***. One of the studies that evaluated α-syn toxicity found that the induction of A53T α-syn expression in PC12 cells increased cell death in a time-dependent manner (Smith et al., 2005). The expression of A53T α-syn inhibited chymotryptic, tryptic, and post-acidic proteasome activity. Additionally, the expression of A53T α-syn significantly increased the intracellular level of reactive oxygen species (ROS). These results indicate that the expression of A53T α-syn in PC12 cells caused early biochemical changes leading to proteasome inhibition and oxidative stress (Smith et al., 2005). The generation of ROS may cause oxidative neuronal damage. The main source of ROS in nigral neurons is thought to be the metabolism of dopamine itself. Another study using SH-SY5Y cells over-expressing human α-syn or mutant A53T or A30P showed increased generation of ROS that was greater in cells expressing α-syn mutants. Furthermore, exposure of all three α-syn-engineered cells to dopamine resulted in decreased cell viability (Junn and Mouradian, 2002).

***Mitochondrial dysfunction***. Mitochondria are membranous organelles that also have been proposed as cellular targets for α-syn neurotoxicity. Release of cytochrome c from mitochondria is a key event inducing apoptosis in many cells including neurons. One study showed that both A53T mutant and WT α-syn were localized at the mitochondria in human neuroblastoma dopaminergic SHSY cells and that overexpression of α-syn A53T or WT in these cells caused the release of cytochrome c from mitochondria (Parihar et al., 2008). The same phenomenon was observed with the induction of A53T α-syn expression in PC12 cells, where the increased release of cytochrome c from the mitochondria and its accumulation in the cytosol was also associated with increased caspase-3 and -9 activities over time [see Figure 3(3)] (Smith et al., 2005).

Mitochondria are main cellular calcium stores and play a crucial role for cellular calcium homeostasis. It is known that alterations in cellular calcium levels contribute to apoptosis of DA neurons in PD. The expression of α-syn A53T or WT is associated with higher concentrations of mitochondrial Ca^2+^, and excess Ca^2+^ initiates an apoptotic pathway. Thus, α-syn induces apoptosis via mitochondria-dependent pathways, including activation of caspases (Parihar et al., 2008).

Another study showed that aggregated and un-aggregated α-syn binds to mitochondrial membranes of human dopaminergic neuroblastoma SHSY cells (Parihar et al., 2009). Significantly higher mitochondrial ROS was observed in cells overexpressing α-syn, known to cause mitochondrial and cellular damage. The level of ROS in A53T α-syn expressing cells was higher than in A30P and WT expressing cells, suggesting greater neuronal toxicity of the A53T mutant. In addition, the mitochondrial membrane potential (Δψ) was measured as an indicator of mitochondrial function. A decrease in Δψ was observed in α-syn overexpressing cells in which A53T expressing cells had the lowest Δψ. These findings suggest that the decreased Δψ and consequent mitochondrial dysfunction are more likely to be a result of mitochondrial damage by elevated mitochondrial ROS and Ca^2+^ (Parihar et al., 2009).

***ER stress***. Cellular accumulation of misfolded proteins can lead to chronic endoplasmic reticulum stress (ERS) and trigger an integrated cellular response called unfolded protein response (UPR) (Doyle et al., 2011) that protects cells from accumulation of toxic misfolded proteins. However, persistent ERS leads to the activation of cell death cascade (Colla et al., 2012a).

A study found an increase in ER chaperones such as grp94, grp78, and PDI (widely used markers of ERS/UPR activation) in affected regions with α-syn pathology using a transgenic (Tg) mouse model expressing human α-syn (WT or mutant). The investigators identified a subset of phosphorylated α-syn reactivity that was localized on the ER membranes. The ER morphology in these neurons was highly abnormal with severely dilated ER cisternae, an ultrastructural indication of ER dysfunction in the A53T α-syn Tg mice (Colla et al., 2012a). Another study examined postmortem tissues of human PD patients and mouse cases of α-synucleinopathy and found selective accumulation of toxic α-syn oligomers within the ER/microsome compartment, supporting a direct pathological link between α-syn oligomers and ER stress *in vivo* (Colla et al., 2012b). The α-synucleinopathy and ER stress in the Tg mice model was also associated with the increased cleavage of caspase-12 and other downstream caspases (Colla et al., 2012a). The cleavage and activation of procaspase-12 is the hallmark of ER stress-induced apoptosis. In PC12 cells, the induction of A53T expression and the activity of caspase-12 was also significantly increased, suggesting that the expression of A53T α-syn causes ER stress mediated apoptosis *in vitro* (Smith et al., 2005).

***Membrane disruption and pore formation***. Another vastly studied cellular toxicity pathway of α-syn is the permeabilization of cellular membranes by oligomers (Stockl et al., 2013). As seen in [Figure 3(2)], the oligomers might interfere with the normal functions of cellular membranes and form pore-like structures, resulting in abnormal calcium influx (or other ions) with consequent neurodegeneration (Tsigelny et al., 2012).

Several studies indicate that α-syn oligomers are able to interact with lipid membranes, increase the conductance and form a pore complex in planar lipid bilayers (Kim et al., 2009; Schmidt et al., 2012; Tosatto et al., 2012). The same effect was observed in HEK293T cells over-expressing α-syn (Tsigelny et al., 2007). It has been reported that mutant α-syn (A53T or A30P) causes higher membrane permeability and induces the formation of pores in the plasma membrane of SH-SY5Y cells, which allows Ca^2+^ influx, and therefore plays an important role in cell degeneration (Furukawa et al., 2006). Similarly, a study by Feng et al. (2010) in a dopaminergic-like cell model demonstrated that the over-expression of WT α-syn caused the formation of oligomeric α-syn pore-like structures. Using whole-cell patch-clamp recordings, an increase in membrane conductance was found indicating the presence of opened membrane channels. These findings were associated with a modest but significant time-dependent increase in cell death and the authors hypothesized that pores formed by α-syn would act as non-selective channels and contribute to α-syn-induced toxicity (Feng et al., 2010).

According to molecular dynamic simulations done by Tsigelny et al. (2012), α-syn monomers penetrate the membrane and via attractive energies of intermolecular contact can develop a ring structure (α-syn octamer). This assembly possesses an intrinsic opening diameter of around 35Å in the middle of the oligomer and an external diameter of 130Å. The investigators observed that the accumulation of α-syn in neuronal membranes altered cellular function. Neurons overexpressing α-syn displayed increased permeability and this effect was more apparent in the cells expressing A53T α-syn. Consistent with this increased permeability, neuronal cell cultures expressing either WT or A53T α-syn displayed increased levels of intracellular calcium. In summary, these results suggest a model where penetration of membranes by α-syn gives rise to the formation of annular pore-like oligomeric structures with the ability to increase cell permeability and calcium influx (Tsigelny et al., 2012).

## Conclusions

Accumulation of misfolded proteins have been linked to the generation and propagation of neurodegenerative diseases such as PD which is characterized by the degeneration and loss of DA neurons in the SNpc that leads to the main motor symptoms of the disease, as well as the degeneration of other non-dopaminergic brain regions. Protein inclusions called Lewy Bodies of which the main component is alpha-synuclein, a pre-synaptic unfolded protein that can adopt different conformations and form aggregates, are found in the cytoplasm of neurons. Mutations in the gene that encodes α-syn (A53T, A30P or E46K) result in proteins with a high tendency to misfold and form fibrillar aggregates rich in β-sheet structure. These mutations highlight the relevance and involvement of α-syn in the onset of PD cases.

Throughout this review we have referred to two important properties of alpha-synuclein. First, the ability of this protein to move from neuron to neuron, propagating the pathology by using a seeding/nucleation mechanism similar to prion proteins where misfolded α-syn recruits endogenous native α-syn to misfold and aggregate. Interestingly, the α-syn transmission occurs through different mechanisms such as exocytosis/endocytosis, by exosomes or directly by penetrating the plasma membrane. Second, the ability of α-syn to cause cellular toxicity, mainly in its oligomeric state. Studies have reported that α-syn oligomers affect several cellular organelles by binding to them and impairing their physiological functions. The most investigated mechanisms of WT and mutated α-syn toxicity include proteasome inhibition, oxidative stress, endoplasmic reticulum stress that leads to the activation of caspases, mitochondrial dysfunction and the eventual release of cytochrome c that also activates caspase pathways. One relevant mechanism of α-syn cytotoxicity is the formation of pore-like structures in different kinds of cellular membranes that act as a non-selective channel allowing the passage of small molecules and cations like Ca^2+^ that are well known to be involved in neurodegeneration. A recent study demonstrated that Aβ also forms pore-like structures and allows the entry of molecules such as Ca^2+^ that could cause neurotoxicity in brains of AD patients (Sepulveda et al., 2014). Table 1 shows the similarities and differences between α-syn and Aβ peptide.

**Table 1:** **Comparison between α-synuclein and β-amyloid characteristics**.

	**α-synuclein**	**B-amyloid**	**References**
Amino acids	140	42	Masters et al., 1985; Bisaglia et al., 2009
Accumulation	Intracellular Lewy body	Extracellular Senile plaques	Soto, 2003
Wide membrane association	Yes	Yes	Sepulveda et al., 2010; Pacheco et al., 2015
Pore formation	Yes	Yes	Feng et al., 2010; Sepulveda et al., 2010
Estimated inner pore diameter	35 Å	15-27 Å	Jang et al., 2007; Tsigelny et al., 2012
Influx of glucose analog (6-NBDG)	Yes	Yes	Sepulveda et al., 2014; Pacheco et al., 2015
Calcium influx	Yes	Yes	Tsigelny et al., 2012; Sepulveda et al., 2014
Most toxic specie	Oligomers	Oligomers	Aguayo et al., 2009; Stockl et al., 2013
Synaptic toxicity	Late ?	Vesicular depletion	Parodi et al., 2010; Pacheco et al., 2015
Cellular transmission	Yes	?	Luk et al., 2012a,b

The reason why α-syn is able to propagate and cause toxicity is not fully understood, but may rely on its conformational plasticity which allows the protein to adopt a secondary structure under different conditions. A study by Danzer et al. (2007) identified diverse types of α-syn oligomers using distinct preparation protocols. One oligomer type increased the intracellular calcium level, provoked increase membrane permeability and triggered cell death. Other oligomers were able to enter cells directly and seed intracellular α-syn aggregation. The conclusion of this study was that depending on the brain milieu conditions, heterogeneous populations of α-syn oligomers could be formed that have different biophysical properties and cellular effects (Danzer et al., 2007). Therefore, some important questions would be, what makes α-syn able to form different aggregated structures that differ in number of monomers and cellular effects? Why does α-syn use a transmission mechanism or cause a toxic effect in one type of cell, and in other cells uses another transmission mechanism and causes other toxic effects? These are questions that would be relevant to discuss in further studies. The properties of α-syn discussed in this review could explain why PD is a progressive and irreversible disease. However, it is possible that α-syn is not the only agent participating in the development and progression of PD, but it does indeed have a strong influence on the complexity of developing an effective therapy for PD.

### Conflict of interest statement

The authors declare that the research was conducted in the absence of any commercial or financial relationships that could be construed as a potential conflict of interest.

## Abbreviations

Aβ: amyloid beta
α-syn: alpha synuclein
A30P: alanine 30 proline
A53T: alanine 53 threonine
AD: Alzheimer's disease
DA: dopamine
E46K: glutamic acid 46 lysine
ER: endoplasmic reticulum
ERS: endoplasmic reticulum stress
Grp 78: glucose-regulated protein 78
Grp 94: glucose-regulated protein 94
LB: Lewy body
L-dopa: L-dihydroxyphenylalanine
LN: Lewy neurite
PD: Parkinson's disease
PDI: protein disulfide isomerase
PFF: preformed fibrils
PrP: prion protein
PrP^C^: cellular prion protein
PrP^SC^: scrapie prion protein
pS129-α-syn: α-syn phosphorylated at serine 129
ROS: reactive oxygen species
Ser129: serine 129
SNARE: soluble *N*-ethylmaleimide-sensitive factor attachment protein receptor
SNpc: substantia nigra pars compacta
Tg: transgenic
TKO: triple knock-out
TNT: tunneling nanotubes
UPR: unfolded protein response
WGA: wheat germ agglutinin
WT: wild type.
